# Real-world 24-month pain outcomes of disk percutaneous ablation and extraction versus Disc-FX nucleoplasty for lumbar discogenic pain and contained lumbar disk herniation: a single-center retrospective cohort study

**DOI:** 10.3389/fneur.2026.1839430

**Published:** 2026-06-23

**Authors:** Xiaohui Yang, Jiaxiang Ni

**Affiliations:** 1 Department of Pain Management, Aerospace Center Hospital, Beijing, China; 2 Department of Pain Management, Capital Medical University Xuanwu Hospital, Beijing, China

**Keywords:** disk percutaneous ablation and extraction, Disc-FX, discogenic pain, lumbar disk herniation, minimally invasive spine surgery, real-world study

## Abstract

**Introduction:**

Discogenic pain and contained lumbar disk herniation are important causes of chronic low back pain. Current intradiscal minimally invasive techniques have long-term efficacy limitations. Disk percutaneous ablation and extraction (DPAE) lacks systematical long-term outcomes evaluation. This study compared mid-to-long-term clinical outcomes of DPAE and standard Disc-FX in such patients.

**Methods:**

This single-center retrospective cohort study included 198 patients: 110 underwent DPAE, 88 received Disc-FX. Primary outcome was the change in low back pain visual analog scale (VAS) score at 24 months. Secondary outcomes included Oswestry Disability Index (ODI), Patient Global Impression of Change (PGIC), achievement of the minimal clinically important difference (MCID), composite treatment success, and reintervention rate. The safety endpoint was perioperative/follow-up complications. Propensity score matching controlled confounding; statistical analyses included analysis of covariance, linear mixed-effects models, logistic regression, and Cox models.

**Results:**

At 24 months, DPAE group had a mean VAS reduction of 5.29 ± 2.06 vs. 4.17 ± 2.24 in Disc-FX group (*p* < 0.001), with an additional 1.05-point reduction (*β* = −1.05, 95%CI −1.57 to −0.53; *p* < 0.001). The DPAE group demonstrated better improvement in ODI, higher PGIC marked improvement, higher VAS/ODI MCID rates, and higher composite success rate (70.0% vs. 51.1%, all *p* < 0.05). Reintervention rate was 7.3% (DPAE) vs. 14.8% (Disc-FX); DPAE reduced reintervention risk (HR = 0.46, 95%CI 0.20 to 0.99; *p* = 0.047). Complication rates (6.4% vs. 9.1%) had no statistically significant difference.

**Discussion:**

Disk percutaneous ablation and extraction may offer a more durable minimally invasive treatment option. Future multicenter prospective studies are warranted to further validate its long-term efficacy and define its optimal indications.

## 1 Introduction

Discogenic pain is a diagnostically challenging and predominant source of chronic LBP (1). It arises from a complex pathophysiology beyond simple degeneration, involving structural failure (disk dehydration, annular fissures, herniation) coupled with inflammatory amplification and neoinnervation (2). Mechanistic studies implicate TNF-α, NF-κB, and oxidative stress pathways (3). MRI is key for identifying pain generators like annular defects and endplate changes (4). Consequently, intradiscal therapy has evolved from simple decompression to multi-mechanistic strategies addressing pressure, fissures, nerve ingrowth, and inflammation (5).

Evidence supports intradiscal interventions. Systematic reviews confirm thermal annular modulation provides significant pain relief up to 12 months (6), with RCTs showing biacuplasty superior to sham (7) and adding value to conservative care (8). Meta-analyses demonstrate ozone injection outperforms steroids (9), condoliase shows ~78% efficacy (10), and PRP improves long-term outcomes (11). These findings suggest multi-mechanistic approaches represent a key evolution.

Among current techniques, Disc-FX is a widely used nucleo-annuloplasty combining limited nucleotomy with RF ablation and modulation. It demonstrates favorable outcomes and low complication rates (12, 13). However, a 7-year follow-up revealed variable long-term durability and persistent reoperation risk influenced by patient factors (14). A pilot study showing limited differences versus another decompression technique suggests potential constraints of single-mechanism approaches, highlighting the need for more integrated strategies (15).

DPAE has emerged as a multi-mechanistic strategy. Unlike Disc-FX, DPAE integrates mechanical nucleotomy, low-temperature ablation (plasma or electromagnetic), annular fissure modulation, ablation of nociceptive nerves/granulation tissue, and inflammatory mediator clearance within a single percutaneous channel. It may also address focal calcifications. This multi-targeted approach aims for synergistic effects on pain generation, potentially offering a more comprehensive treatment. While full-endoscopic techniques are effective, they entail a steeper learning curve and higher procedural demands compared to this more streamlined intradiscal approach (16). Thus, DPAE may offer a favorable balance of minimal invasiveness, mechanistic breadth, and accessibility, particularly for discogenic pain or contained herniations in patients unsuitable for or averse to surgery.

This study therefore compares 24-month real-world outcomes of DPAE versus standard Disc-FX in patients with chronic discogenic LBP and contained herniations, assessing pain relief, functional recovery, clinical success, reintervention risk, and safety to provide robust long-term evidence for optimizing treatment selection.

## 2 Materials and methods

### 2.1 Patient data

#### 2.1.1 Study design

This study was designed as a retrospective single-center cohort investigation. Eligible patients were identified from a prospectively maintained clinical database at the Spine Minimally Invasive Center of a tertiary hospital. This database systematically records baseline clinical characteristics, imaging findings, procedural details, and longitudinal follow-up information for all patients undergoing percutaneous disk interventions at our institution. The enrollment period extended from June 2017 to December 2021. During this interval, 256 patients underwent percutaneous minimally invasive disk procedures for chronic lumbar discogenic pain or contained lumbar disk herniation. After application of the predefined eligibility criteria, 198 patients were included in the final analysis.

All included patients were diagnosed with lumbar discogenic pain or contained lumbar disk herniation based on a comprehensive clinical assessment integrating presenting symptoms, physical examination findings, and imaging features. Before surgery, all patients had received systematic conservative treatment, including pharmacological therapy, physical rehabilitation, or other non-surgical interventions, but continued to experience persistent or recurrent symptoms that significantly impaired daily activities and quality of life.

Patients were allocated into two groups according to the minimally invasive procedure performed. The DPAE group consisted of 110 patients who underwent Disc Percutaneous Ablation and Extraction, a technique combining mechanical forceps extraction with low-temperature electromagnetic wave ablation. The Disc-FX group comprised 88 patients who underwent standard Disc-FX radiofrequency ablation. Both procedures are established minimally invasive intradiscal interventions routinely used at our institution.

To reduce potential confounding related to operator variability, all procedures were performed by the same dedicated spine minimally invasive surgical team. This team has extensive experience in percutaneous disk interventions and follows uniform technical protocols and standardized perioperative management pathways. Clinical data were extracted from the hospital electronic medical record system and follow-up database. Data extraction was independently performed by two investigators and subsequently cross-checked to ensure accuracy and completeness.

#### 2.1.2 Ethics and informed consent

The study was reviewed and approved by the ethics committee of the Aerospace Center Hospital (2021-ASCH-013). All research procedures conformed to the ethical standards set forth in the Declaration of Helsinki for studies involving human participants. Owing to the retrospective nature of the investigation, the data analyzed were derived from routinely documented clinical records. To protect patient privacy, all identifiable information was removed prior to data collection and statistical analysis, and the dataset was used solely for scientific research purposes.

### 2.2 Eligibility criteria

#### 2.2.1 Inclusion criteria

1. Age between 18 and 75 years, regardless of gender.
2. Confirmed diagnosis of chronic lumbar discogenic pain or contained lumbar disk herniation based on integrated clinical evaluation, including: (1) characteristic axial low back pain with or without mild radicular symptoms; (2) positive clinical signs such as localized tenderness or pain on provocative maneuvers; (3) imaging evidence from MRI showing disk degeneration, annular fissures, or contained herniation; and (4) optional confirmation by discography when clinical and imaging findings are inconclusive.
3. Symptom duration of at least 6 months, with clinical manifestations predominantly consisting of chronic axial low back pain, with or without mild-to-moderate radicular irritation.
4. Imaging findings consistent with symptomatic disk pathology at the corresponding level. MRI had to demonstrate disk degeneration or contained herniation, including patients with annular tears (annular fissures), with concordance between imaging abnormalities and clinical symptoms or signs.
5. Failure of standardized conservative treatment for at least 3 months, including medication, physical therapy, rehabilitation, or other non-surgical interventions.
6. Predominantly axial low back pain with VAS ≥ 4, with or without mild-to-moderate radicular symptoms, but without evidence of progressive neurological deficit.
7. Single-level or two-level lumbar disk disease.
8. Underwent either DPAE or Disc-FX minimally invasive disk intervention and completed at least 24 months of postoperative follow-up.

#### 2.2.2 Exclusion criteria

1. Extruded or sequestered disk herniation, or any other concurrent pain source such as lumbar facet joint pain, sacroiliac joint pain, radicular pain from nerve root compression, or lumbar myofascial pain, especially if likely to interfere with 24-month follow-up assessment.
2. Imaging evidence of clinically significant lumbar spinal stenosis, lateral recess stenosis, or foraminal stenosis as the primary source of symptoms.
3. Significant lumbar instability, including spondylolisthesis of grade II or higher, or abnormal vertebral motion on dynamic radiographs.
4. Prior open lumbar surgery or fusion at the symptomatic level(s).
5. Concurrent spinal infection, spinal tumor, vertebral fracture, or other major structural spinal pathology.
6. Severe neurological deficit, progressive neurological deterioration, or cauda equina syndrome requiring urgent surgical treatment, or patients who develop new non-discogenic pain requiring intervention during follow-up were excluded from per-protocol analysis.
7. Severe systemic or inflammatory disease, including significant cardiopulmonary insufficiency, coagulopathy, or other conditions rendering the patient unsuitable for the procedure.
8. Incomplete clinical records or insufficient follow-up data precluding reliable outcome assessment.

### 2.3 Study methodology and group allocation

#### 2.3.1 Surgical techniques

All procedures were performed in a standard sterile operating room under real-time C-arm fluoroscopic guidance. Patients were placed in a prone position with a pillow under the abdomen to reduce lumbar lordosis and facilitate posterolateral access, thereby lowering intradiscal pressure. Prophylactic intravenous antibiotics were administered prior to the procedure, and mild intravenous sedation was provided as needed. After routine sterile preparation and draping, all interventions were performed under local infiltration anesthesia. A posterolateral transforaminal approach was consistently used, with access to the target disk obtained through Kambin’s safety triangle. Patients were categorized into the DPAE or Disc-FX group according to the specific minimally invasive technique performed.

##### 2.3.1.1 DPAE group

DPAE is a multimodal percutaneous intradiscal intervention integrating mechanical decompression, low-temperature electromagnetic wave ablation, and annular modulation through a single percutaneous working channel. The procedure began with fluoroscopic confirmation of the target disk level. A 17-gauge spinal needle was used for puncture under local infiltration anesthesia with 1% lidocaine, combined with mild intravenous sedation. The puncture trajectory and insertion angle were determined using anteroposterior and lateral fluoroscopic views, with the C-arm squared to the endplate (square the end plate) and rotated 30°–45° to obtain oblique images for optimal posterolateral access. The skin entry point was located 8–12 cm lateral to the midline. The needle was carefully advanced to reach the center of the nucleus pulposus, and final tip placement within the disk was confirmed on both anteroposterior and lateral fluoroscopic images. Optional sensory and motor stimulation (0.5–2 mA) was applied to confirm safe proximity to neural structures.

After confirmation of correct needle placement, a guidewire was inserted and the puncture needle was removed. Sequential dilators were advanced over the guidewire to establish a stable percutaneous tract. A working cannula (5.5 mm internal diameter) was introduced, with its distal tip positioned within the disk space. Repeated mechanical nucleotomy was performed using specialized disk grasping forceps until intradiscal resistance decreased and no additional tissue could be removed, providing an objective indication of reduced intradiscal pressure.

After mechanical nucleotomy, a flexible low-temperature electromagnetic ablation electrode (DPAE Generator, SpineTech, Model X5, United States) was inserted. Ablation was performed at 32–36 °C for 60 s per target region, not exceeding 42 °C, and the electrode curvature was adjusted to selectively target degenerated nucleus, granulation tissue, and nociceptive nerve endings.

The electrode was then redirected to ablate the posterior annular fissure. At the conclusion, 2 mL of 1% lidocaine mixed with 10 mg triamcinolone was injected into the posterior annulus via the working channel. Proper placement was verified fluoroscopically, and the cannula was withdrawn after ensuring hemostasis and absence of nerve irritation. Representative intraoperative fluoroscopic images of DPAE are shown in Supplementary Figure S1.

##### 2.3.1.2 Disc-FX group

Patients assigned to the Disc-FX group were treated with standard Disc-FX radiofrequency-assisted nucleo-annuloplasty (Disc-FX Generator, SpineTech, Model FX-2, United States). The procedure was performed under fluoroscopic guidance through a posterolateral transforaminal route, with puncture via Kambin’s safety triangle. A 17-gauge spinal needle was used for disk access, followed by insertion of a 5.5 mm internal diameter working cannula. Limited mechanical nucleotomy was performed using Disc-FX instruments to reduce intradiscal pressure and remove only degenerated nucleus material while preserving structural integrity of the disc, thus minimizing risk of disk collapse or instability. Optional sensory and motor stimulation (0.5–2 mA) confirmed safe proximity to neural structures.

Radiofrequency ablation was applied at 40–45 °C for 60 s per lesion, with 2–3 lesions placed in the nucleus and posterior annulus as needed. At the conclusion of the procedure, 2 mL of 1% lidocaine mixed with 10 mg triamcinolone was injected into the posterior annulus to reduce postoperative inflammation and provide early analgesia. Fluoroscopic images confirming proper cannula and electrode placement are shown in Supplementary Figure S2.

All patients in both groups received standardized postoperative care. This included a regimen of oral nonsteroidal anti-inflammatory drugs (NSAIDs) such as ibuprofen 400 mg every 8 h for 5 days, and acetaminophen 500 mg as needed for breakthrough pain. Early mobilization was encouraged on the day of surgery, with gradual return to normal daily activities over the following week. Patients were observed in the outpatient day-surgery unit for 4–6 h postoperatively before discharge, confirming hemodynamic stability, absence of neurological deficit, and adequate pain control.

#### 2.3.2 Variable collection and definitions

Baseline information was retrieved from the hospital’s electronic medical records and institutional clinical registry. The demographic characteristics collected for analysis included patient age, gender, and body mass index. Clinical history variables included symptom duration, previous conservative treatment history, and symptomatic level distribution. Imaging variables were derived from preoperative lumbar magnetic resonance imaging examinations.

All MRI scans were independently reviewed by two experienced spine radiologists. In cases of disagreement, a consensus interpretation was reached through discussion. Disk degeneration severity was graded using the Pfirrmann classification, which categorizes degeneration from grade I to grade V according to nucleus signal intensity, disk structure, and intervertebral disk height. Symptomatic level(s) were determined by integrating imaging abnormalities with the patient’s clinical presentation and physical examination findings.

Baseline evaluations before surgery included both pain severity and functional impairment. Pain severity was quantified with the visual analog scale (VAS), which uses a 0-to-10 continuum, with 0 representing the absence of pain and 10 representing the most severe pain perceived by the patient. Functional capacity was evaluated using the Oswestry Disability Index (ODI).

Patients were followed postoperatively at predefined intervals of 1, 3, 6, 12, and 24 months after the procedure. Follow-up assessments were completed by a dedicated follow-up team through outpatient visits or telephone interviews. Data on clinical recovery and related outcomes were collected in a standardized manner and entered into the follow-up database.

### 2.4 Outcome measures

To systematically compare the clinical efficacy of DPAE and Disc-FX in terms of pain relief, functional recovery, and long-term safety, predefined primary and secondary outcomes were assessed at standardized follow-up time points. The primary outcome was the change in low back pain, measured using the VAS from baseline to 24 months. Patients’ pain intensity was recorded at baseline and at 1, 3, 6, 12, and 24 months, with the 24-month change serving as the primary endpoint. Secondary outcomes included functional recovery evaluated with the Oswestry Disability Index (ODI), patient-perceived improvement assessed by the Patient Global Impression of Change (PGIC), achievement of the minimal clinically important difference (MCID) for pain and function, composite treatment success, and reintervention rates. Safety outcomes encompassed perioperative and follow-up procedure-related complications, which were systematically documented and classified according to standardized criteria. All outcomes were collected by trained personnel using uniform procedures to ensure consistency and reliability.

### 2.5 Statistical analysis

Statistical analyses were performed using R and SPSS 26.0. Continuous variables were summarized as mean ± standard deviation or median (IQR) and compared using *t*-tests or Mann–Whitney *U* tests, as appropriate. Categorical data were expressed as number (%) and compared with chi-square or Fisher’s exact tests. To reduce confounding in this non-randomized study, 1:1 propensity score matching was performed using a logistic regression model including age, gender, BMI, symptom duration, disk level, Pfirrmann grade, and baseline VAS/ODI. Balance was assessed using standardized mean differences (SMD < 0.10).

Primary outcome (VAS change at 24 months) was analyzed with ANCOVA adjusting for baseline covariates. Repeated measures were analyzed using linear mixed-effects models. Binary outcomes (MCID achievement, composite success, PGIC) were evaluated using logistic regression. Time-to-event outcomes (reintervention) were assessed by Kaplan–Meier curves and Cox proportional hazards models.

Complications were classified according to the Clavien-Dindo grading system: grades I–II for minor events resolving with conservative management, grade ≥III for events requiring surgical, endoscopic, or prolonged interventions. *p* < 0.05 was considered statistically significant.

## 3 Results

### 3.1 Patient flow and baseline characteristics

**Figure 1:**
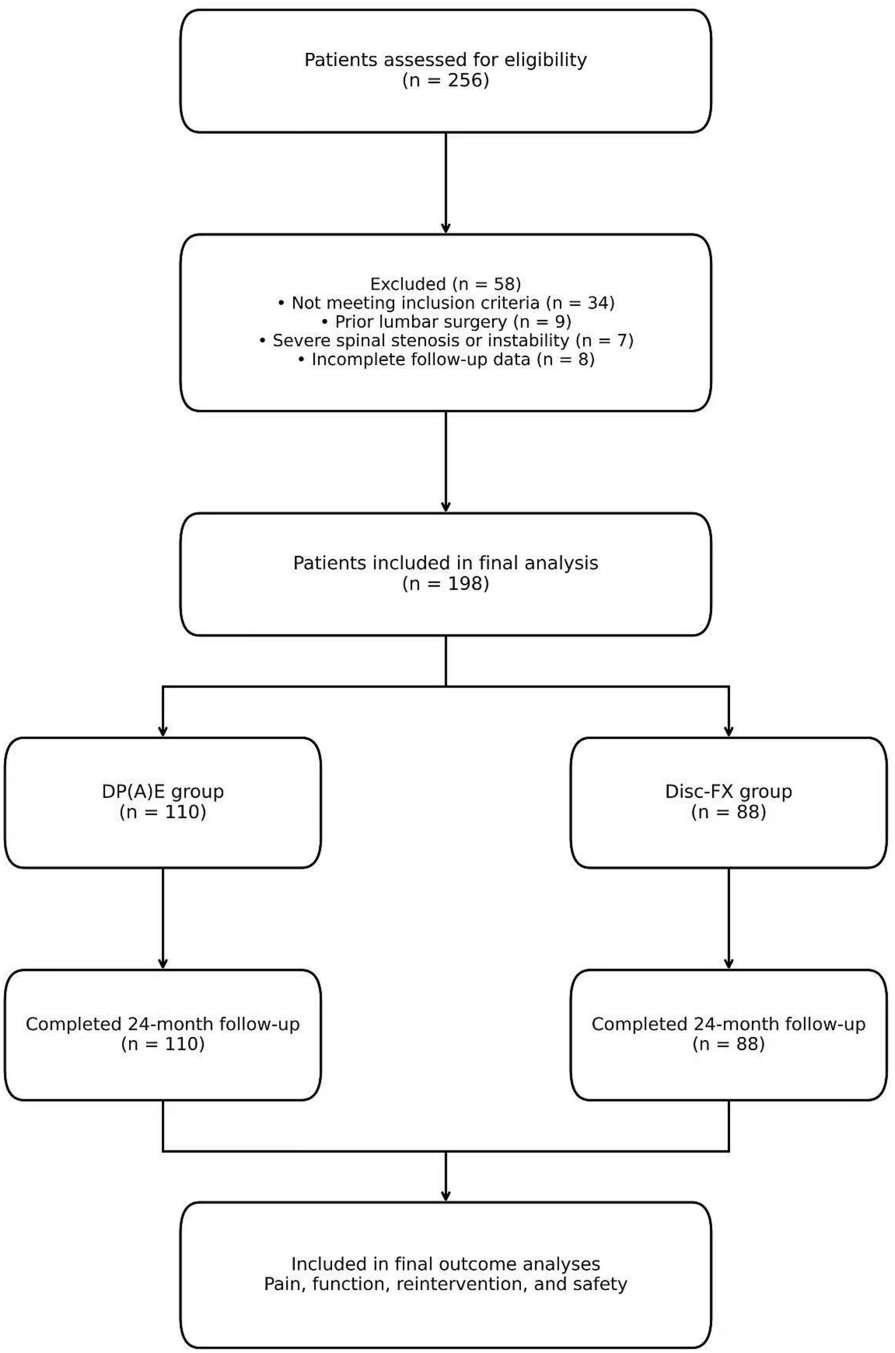
Flowchart of patient screening and study inclusion.

A total of 256 patients who underwent percutaneous minimally invasive disk interventions for chronic lumbar discogenic pain or contained herniation were screened. After applying predefined inclusion and exclusion criteria, 198 patients were enrolled: 110 in the DPAE group and 88 in the Disc-FX group. The selection process is shown in Figure 1.

Before PSM, baseline demographic and clinical characteristics were comparable between the two groups. No significant differences were observed in age, gender distribution, body mass index, preoperative pain intensity (VAS), functional disability (ODI), symptom duration, affected disk level, number of levels, or Pfirrmann degeneration grade (all *p* > 0.05, Table 1).

**Table 1:** Baseline characteristics before and after propensity score matching.

Variable	DP(A)E (*n* = 110)	Disc-FX (*n* = 88)	*p*-value	SMD	DP(A)E (*n* = 82)	Disc-FX (*n* = 82)	*P*-value	SMD
Age, years	66.8 ± 13.5	67.3 ± 14.0	0.79	0.04	66.9 ± 13.2	67.1 ± 13.6	0.91	0.02
Female, *n* (%)	60 (54.5)	50 (56.8)	0.75	0.05	45 (54.9)	46 (56.1)	0.88	0.02
BMI, kg/m^2^	24.6 ± 2.9	24.8 ± 2.7	0.61	0.07	24.7 ± 2.8	24.8 ± 2.6	0.84	0.03
Symptom duration, months	13 (8–18)	14 (9–18)	0.48	0.08	13 (8–17)	13 (9–17)	0.81	0.03
Baseline VAS score	7.34 ± 1.82	7.29 ± 1.89	0.84	0.03	7.31 ± 1.80	7.30 ± 1.84	0.96	0.01
Baseline ODI score	45.6 ± 8.4	46.2 ± 8.6	0.62	0.07	45.8 ± 8.3	45.9 ± 8.5	0.94	0.01
Smoking status			0.71	0.06			0.89	0.03
Never	70 (63.6)	58 (65.9)			52 (63.4)	53 (64.6)		
Former	26 (23.6)	19 (21.6)			19 (23.2)	18 (22.0)		
Current	14 (12.8)	11 (12.5)			11 (13.4)	11 (13.4)		
Hypertension	48 (43.6)	40 (45.5)	0.79	0.04	36 (43.9)	37 (45.1)	0.87	0.02
Diabetes mellitus	15 (13.6)	13 (14.8)	0.82	0.03	11 (13.4)	12 (14.6)	0.84	0.03
Affected level			0.85	0.07			0.96	0.04
L2–3	4 (3.6)	3 (3.4)			3 (3.7)	3 (3.7)		
L3–4	24 (21.8)	20 (22.7)			18 (22.0)	18 (22.0)		
L4–5	85 (77.3)	70 (79.5)			63 (76.8)	65 (79.3)		
L5–S1	18 (16.4)	14 (15.9)			13 (15.9)	13 (15.9)		
Number of affected levels			0.51	0.05			0.88	0.02
Single level	84 (76.4)	69 (78.4)			63 (76.8)	64 (78.0)		
Two levels	26 (23.6)	19 (21.6)			19 (23.2)	18 (22.0)		
Pfirrmann grade			0.73	0.06			0.94	0.03
Grade III	21 (19.1)	16 (18.2)			15 (18.3)	15 (18.3)		
Grade IV	60 (54.5)	49 (55.7)			45 (54.9)	46 (56.1)		
Grade V	29 (26.4)	23 (26.1)			22 (26.8)	21 (25.6)		

Continuous variables are presented as mean ± standard deviation or median (interquartile range), and categorical variables are presented as number (percentage). SMD, standardized mean difference; VAS, visual analog scale; ODI, Oswestry Disability Index; BMI, body mass index. SMD < 0.10 indicates adequate covariate balance after propensity score matching.

After 1:1 PSM (adjusting for age, gender, BMI, symptom duration, level, Pfirrmann grade, baseline VAS and ODI), 82 matched pairs were obtained. All covariates achieved good balance with standardized mean differences below 0.10, indicating successful mitigation of confounding (Table 1).

### 3.2 Primary outcome

#### 3.2.1 Pain reduction at 24 months

At 24 months postoperatively, both groups demonstrated significant pain reduction from baseline. The DPAE group exhibited a significantly greater mean reduction in VAS compared to the Disc-FX group. This difference remained significant after PSM and after adjustment for potential confounders in ANCOVA, with DPAE providing an additional clinically meaningful reduction in pain scores (Table 2).

**Table 2:** Primary pain outcomes at 24 months.

Outcome	DP(A)E	Disc-FX	Between-group difference/effect size	*P*-value
Original cohort analysis				
Baseline VAS score	7.34 ± 1.82	7.29 ± 1.89	*t* = 0.20	0.84
VAS score at 24 months	2.05 ± 1.74	3.12 ± 2.08	Mean difference = −1.07	<0.001
Change in VAS score	5.29 ± 2.06	4.17 ± 2.24	*t* = 3.66	<0.001
Matched cohort analysis				
VAS score at 24 months	2.08 ± 1.71	3.05 ± 2.02	Mean difference = −0.97	0.001
Change in VAS score	5.23 ± 2.01	4.20 ± 2.16	*t* = 3.18	0.002
Adjusted analysis (ANCOVA)				
Treatment effect of DP(A)E	–	Reference	*β* = −1.05 (95% CI −1.57 to −0.53)	<0.001
Model goodness-of-fit	adjusted *R*^2^ = 0.41		*F* = 22.6	<0.001
Clinical significance analysis				
Patients achieving VAS MCID	91 (82.7%)	60 (68.2%)	*χ*^2^ = 5.75	0.016
Adjusted odds ratio for MCID	–	Reference	OR = 2.21 (95% CI 1.15 to 4.23)	0.017

Continuous variables are presented as mean ± standard deviation and categorical variables as number (percentage). VAS, visual analog scale; MCID, minimal clinically important difference. ANCOVA models were adjusted for baseline VAS score, age, gender, body mass index, symptom duration, and Pfirrmann grade.

The proportion of patients achieving the MCID for pain at 24 months was significantly higher in the DPAE group than in the Disc-FX group. Multivariable logistic regression confirmed that DPAE was associated with higher odds of achieving clinically meaningful pain relief (Table 2).

#### 3.2.2 Longitudinal pain trends

**Figure 2:**
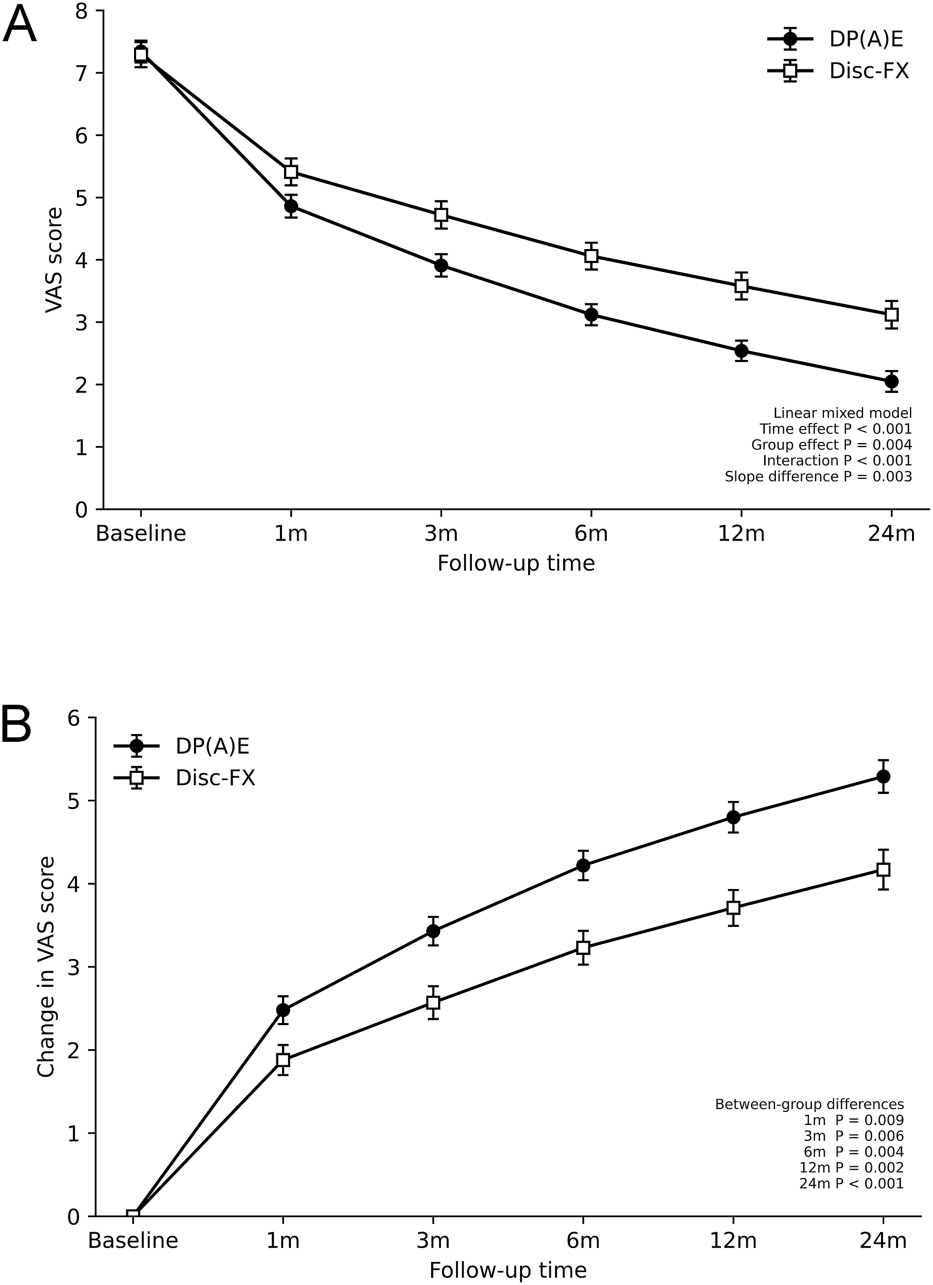
Longitudinal changes in low back pain visual analog scale scores during postoperative follow-up in the two treatment groups. **(A)** Trajectories of VAS scores in the DPAE and Disc-FX groups at baseline and at 1, 3, 6, 12, and 24 months after surgery. **(B)** Comparison of improvement in VAS scores from baseline at each postoperative follow-up time point.

Throughout the 24-month follow-up, VAS scores progressively decreased in both groups. However, the DPAE group consistently demonstrated lower pain scores at all postoperative time points and a steeper improvement trajectory (Figure 2A). The magnitude of pain reduction from baseline was significantly greater in the DPAE group at each follow-up interval (all *p* < 0.01, Figure 2B). Linear mixed-effects modeling revealed significant time and treatment group effects, with a significant time-by-treatment interaction, confirming the superior and more rapid pain relief afforded by DPAE.

### 3.3 Secondary outcomes

#### 3.3.1 Functional recovery

**Figure 3:**
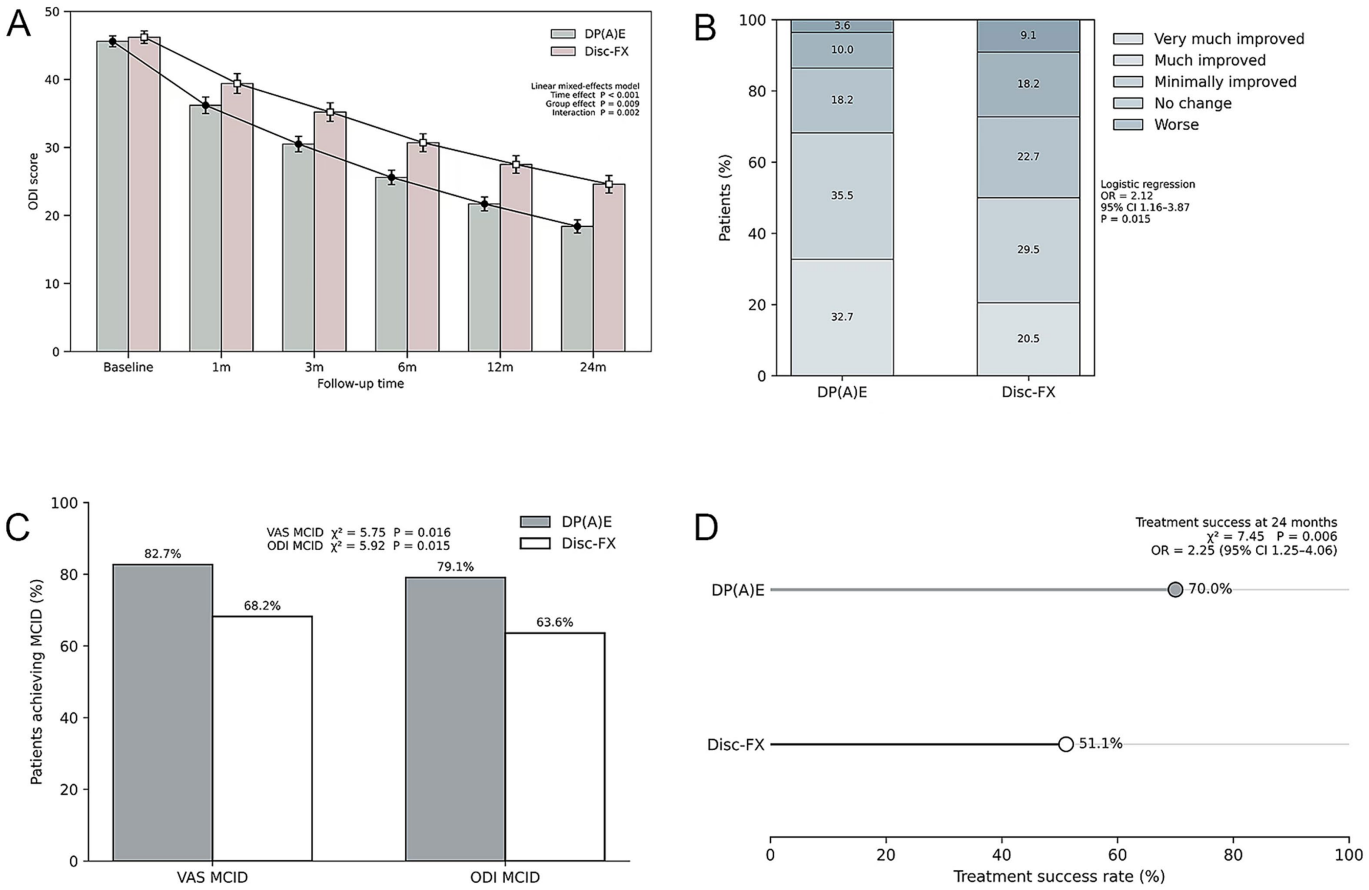
Comparison of functional recovery, patient-perceived global improvement, and clinical success between the two groups. **(A)** Changes in Oswestry Disability Index scores from baseline through postoperative follow-up. **(B)** Distribution of Patient Global Impression of Change scores at 24 months after surgery. **(C)** Comparison of the proportions of patients achieving the minimal clinically important difference. **(D)** Comparison of composite treatment success rates.

Functional status, assessed by ODI, improved significantly in both groups over the 24-month follow-up. The DPAE group demonstrated consistently greater functional improvement than the Disc-FX group at all time points (Figure 3A). At 24 months, the mean ODI improvement was significantly larger in the DPAE group. Linear mixed-effects modeling confirmed significantly superior functional recovery trajectory for DPAE, with significant group and time-by-treatment interaction effects.

#### 3.3.2 Patient global impression of change

At the 24-month assessment, a significantly greater proportion of patients in the DPAE group rated their condition as either “much improved” or “very much improved” on the PGIC scale than those in the Disc-FX group (Figure 3B). This association remained significant in multivariable logistic regression analysis, which showed that DPAE independently increased the likelihood of a favorable overall perception of improvement.

#### 3.3.3 MCID achievement rates

The proportion of patients achieving MCID for pain (VAS-MCID) and for function (ODI-MCID) at 24 months was significantly higher in the DPAE group than in the Disc-FX group (both *p* < 0.05, Figure 3C).

#### 3.3.4 Composite treatment success

The composite treatment success rate (defined as simultaneous achievement of VAS reduction ≥2, ODI improvement ≥10, and no reintervention during follow-up) at 24 months was significantly higher in the DPAE group compared to the Disc-FX group (Figure 3D). Multivariable logistic regression confirmed DPAE was associated with significantly higher odds of achieving composite success.

### 3.4 Reintervention analysis

**Figure 4:**
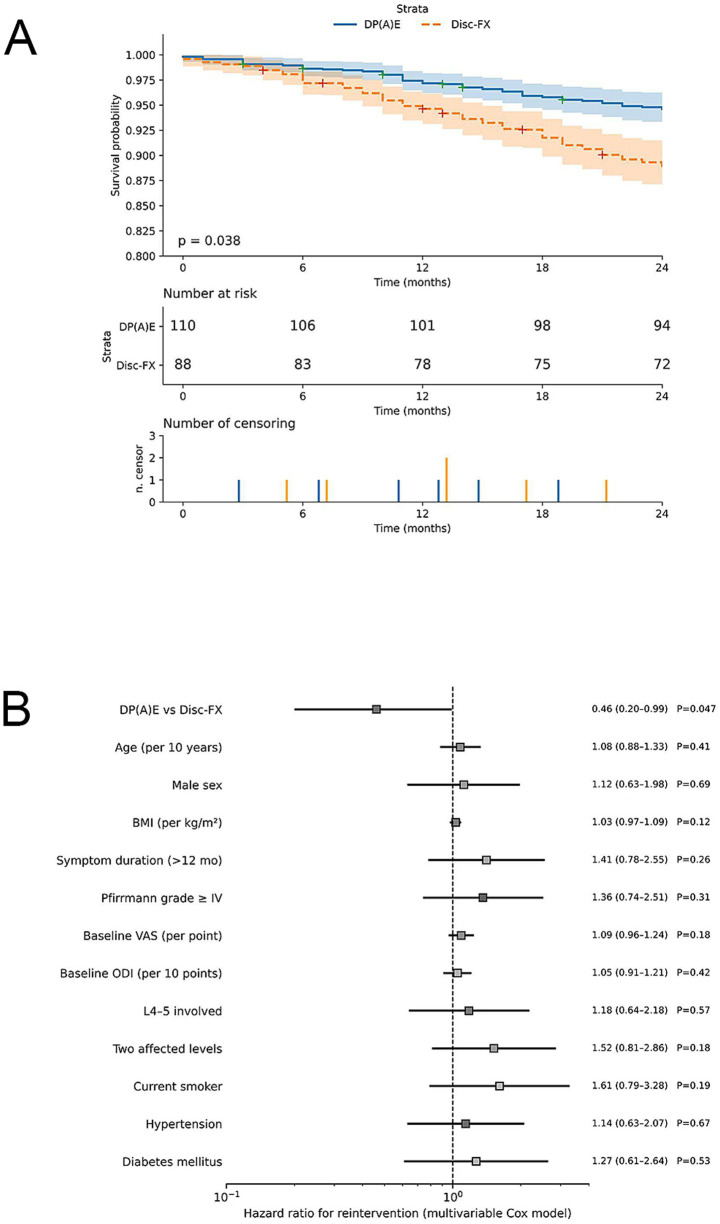
Comparison of reintervention risk between the two treatment strategies. **(A)** Kaplan–Meier curves for reintervention-free survival. **(B)** Forest plot from the multivariable Cox proportional hazards model.

Over the 24-month follow-up period, patients treated with DPAE experienced fewer reintervention events than those in the Disc-FX group, and the difference reached statistical significance (*p* = 0.048). Time-to-event analysis further showed that the probability of remaining free from reintervention was consistently higher in the DPAE group during follow-up (log-rank *p* = 0.038; Figure 4A). After adjustment for relevant baseline covariates, multivariable Cox proportional hazards analysis indicated that treatment with DPAE was independently associated with a lower hazard of reintervention compared with Disc-FX (HR = 0.46, 95% CI 0.20–0.99, *p* = 0.047; Figure 4B).

### 3.5 Safety outcomes

Overall complication rates were low and comparable between the two groups, with no significant difference in the incidence of procedure-related adverse events. Most complications were transient, mild (Clavien-Dindo grade I or II), and resolved with conservative management. No severe complications (permanent neurological deficit, dural injury, major vascular injury, or need for emergency surgery) occurred in either group (Table 3).

**Table 3:** Perioperative and follow-up safety outcomes.

Safety outcome	DP(A)E (*n* = 110)	Disc-FX (*n* = 88)	Effect estimate/test statistic	*P*-value
Overall safety profile				
Any procedure-related complication	7 (6.4%)	8 (9.1%)	OR = 0.68 (95% CI 0.23–2.03)	0.48
Early complications within 30 days	7 (6.4%)	8 (9.1%)	OR = 0.68 (95% CI 0.23–2.03)	0.48
Late complications after 30 days	0	0	–	–
Time to complication onset, days	9 (4–17)	11 (5–21)	*Z* = −0.55	0.58
Complete resolution by final follow-up	7 (100.0%)	8 (100.0%)	Fisher	>0.99
Complication-related readmission	0	1 (1.1%)	Fisher	0.26
Complication-related reoperation	0	0	–	–
Specific adverse events				
Transient nerve root irritation	4 (3.6%)	4 (4.5%)	OR = 0.79 (95% CI 0.19–3.32)	0.75
Superficial wound infection	1 (0.9%)	1 (1.1%)	Fisher	0.89
Discitis	0	1 (1.1%)	Fisher	0.26
Transient postoperative back pain exacerbation	2 (1.8%)	2 (2.3%)	OR = 0.80 (95% CI 0.11–5.84)	0.81
Persistent sensory or motor deficit	0	0	–	–
Dural sac injury	0	0	–	–
Major vascular injury	0	0	–	–
Emergency open surgery	0	0	–	–
Management and clinical course				
Conservative treatment only	7 (6.4%)	7 (8.0%)	*χ*^2^ = 0.18	0.67
Intravenous or prolonged antibiotic therapy	0	1 (1.1%)	Fisher	0.26
Residual symptoms at last follow-up	0	0	–	–
Clavien–Dindo grade				
Grade I	4 (3.6%)	5 (5.7%)		
Grade II	3 (2.7%)	3 (3.4%)	Fisher	0.62
Grade ≥ III	0	0	–	–

Values are presented as *n* (%) or median (interquartile range). Comparisons between groups were performed using the *χ*^2^ test, Fisher exact test, or Mann–Whitney *U* test as appropriate. Complication severity was classified according to the Clavien–Dindo grading system.

### 3.6 Subgroup and heterogeneity analyses

Prespecified subgroup analyses based on 24-month VAS improvement consistently demonstrated superior pain relief with DPAE compared to Disc-FX across all examined subgroups, including age, gender, symptom duration, Pfirrmann grade, affected level, and baseline pain severity. No significant treatment-by-subgroup interactions were detected, indicating that the superior efficacy of DPAE was consistent across diverse patient characteristics (Table 4). The predicted probability of achieving composite treatment success under typical patient characteristics was substantially higher for DPAE than for Disc-FX, with an absolute risk difference exceeding 17%.

**Table 4:** Subgroup and treatment effect heterogeneity analyses.

Analysis domain	Subgroup	DP(A)E	Disc-FX	Between-group effect estimate	*P*-value	*P* for interaction
Change in VAS score at 24 months	Overall	5.29 ± 2.06	4.17 ± 2.24	1.12 (0.52 to 1.72)	<0.001	–
Age	<50 years	5.58 ± 1.95	4.36 ± 2.08	1.22 (0.18 to 2.26)	0.022	0.41
	≥50 years	5.24 ± 2.08	4.14 ± 2.27	1.10 (0.44 to 1.76)	0.001	0.41
Gender	Male	5.21 ± 2.01	4.08 ± 2.18	1.13 (0.33 to 1.93)	0.006	0.78
	Female	5.35 ± 2.11	4.24 ± 2.29	1.11 (0.34 to 1.88)	0.005	0.78
Symptom duration	≤12 months	5.63 ± 1.92	4.31 ± 2.05	1.32 (0.63 to 2.01)	<0.001	0.16
	>12 months	5.04 ± 2.12	4.08 ± 2.31	0.96 (0.24 to 1.68)	0.009	0.16
Pfirrmann grade	I–III	5.47 ± 1.88	4.40 ± 2.01	1.07 (0.08 to 2.06)	0.034	0.69
	IV–V	5.25 ± 2.10	4.12 ± 2.29	1.13 (0.48 to 1.78)	0.001	0.69
Affected level	L4–5	5.34 ± 2.03	4.19 ± 2.21	1.15 (0.51 to 1.79)	<0.001	0.82
	Other levels	5.11 ± 2.17	4.08 ± 2.34	1.03 (0.02 to 2.04)	0.046	0.82
Baseline pain severity	VAS < 7	4.88 ± 1.86	3.92 ± 2.01	0.96 (0.19 to 1.73)	0.015	0.37
	VAS ≥ 7	5.53 ± 2.14	4.33 ± 2.29	1.20 (0.51 to 1.89)	0.001	0.37
Marginal predicted probability of composite treatment success	Typical patient profile	72.4%	54.8%	Absolute risk difference 17.6% (95% CI 6.4 to 28.3%)	0.003	–
				Adjusted OR 2.25 (95% CI 1.25 to 4.06)	0.007	–

Values for subgroup analyses are presented as mean ± standard deviation unless otherwise indicated. Between-group effect estimates represent mean differences in 24-month VAS improvement with 95% confidence intervals. *P* for interaction was derived from multivariable linear regression models including treatment-by-subgroup interaction terms. The marginal predicted probability of composite treatment success was estimated from a multivariable logistic regression model.

## 4 Discussion

Discogenic pain and contained lumbar disk herniation represent challenging chronic LBP subtypes, driven by multifaceted pathophysiology: abnormal intradiscal pressure, annular fissures, local inflammation, and neoinnervation. Conservative care often fails to provide sustained relief, while open surgery is not always indicated for predominantly axial pain with limited imaging findings. Thus, minimally invasive intradiscal interventions targeting multiple pain generators simultaneously are a key research focus. This real-world, propensity-matched cohort study compared 24-month outcomes of DPAE versus standard Disc-FX in these patients. The results demonstrate that DPAE, a multi-mechanistic strategy, provided superior pain relief, functional recovery, clinical success, and lower reintervention risk without increased complications, strongly supporting its clinical utility.

The most notable finding is that DPAE’s advantage was not confined to a single outcome but consistently spanned pain intensity, function, patient global impression, MCID achievement, composite success, and reintervention. This multidimensional benefit, sustained over 24 months, suggests DPAE addresses multiple pain-generating mechanisms within the disc, rather than merely providing transient symptom relief. Methodologically, the convergence of benefits across different outcome hierarchies—from continuous variables (VAS, ODI) to clinically meaningful metrics (MCID, composite success) and durable endpoints (reintervention)—reinforces the robustness and internal consistency of the findings. Recent studies on combined intradiscal procedures for discogenic pain, such as thermocoagulation plus targeted annular modulation, report superior outcomes compared to single-target interventions (17), supporting our conclusion that multi-mechanistic strategies provide more sustained clinical benefits. While full-endoscopic techniques can be effective for radicular symptoms, they require steeper learning curves and higher procedural demands, and are not directly comparable to patients with predominantly discogenic low back pain (2, 6–8). Therefore, these techniques were not considered primary comparators in this study. In contrast, DPAE offers a minimally invasive, multi-targeted intradiscal approach specifically designed for discogenic pain, allowing simultaneous modulation of key pathological processes within the disc.

Although DPAE does not use implants, its thermal modulation of fissure areas may partially stabilize the annulus and reduce local stress and irritation. Furthermore, combination therapies (e.g., ablation plus collagenase) have demonstrated superior pain relief and inflammation reduction compared to ablation alone (18), suggesting that multi-pronged clearance of degenerated tissue and inflammatory mediators is biologically rational. Thus, DPAE’s long-term benefits likely arise from synergistic effects: reducing mechanical load via tissue removal, ablating nociceptive nerve endings and granulation tissue, modulating annular fissures, and lowering inflammatory burden—all contributing to sustained clinical improvement.

In the present study, DPAE achieved superior long-term clinical outcomes without introducing a higher overall risk profile than Disc-FX, a finding of clear relevance to its potential clinical scalability. For any minimally invasive disc-based intervention, an efficacy advantage is of limited practical value if it comes at the expense of increased complications, greater dependence on highly specialized technical pathways, or substantially higher procedural complexity. In this context, the observation that DPAE was associated with better pain relief, functional recovery, and reintervention outcomes while maintaining a comparable safety profile suggests that its therapeutic benefit is unlikely to be driven by more aggressive tissue disruption or a greater perioperative burden. Rather, its advantage may stem from optimization of the treatment mechanism itself. Previous studies on minimally invasive disk procedures, such as transforaminal endoscopic lumbar discectomy (TELD) and percutaneous endoscopic transforaminal discectomy (PETD), have shown favorable outcomes for lumbar discogenic pain, with differences mainly in technical complexity and learning curves. However, these studies focused on radicular pain or herniated disk cases and are not directly comparable to patients with primarily discogenic low back pain; thus, they were not used as primary comparators in this analysis. Consecutive case series and retrospective studies have likewise confirmed that full-endoscopic discectomy is generally safe and effective, yet its long-term results remain strongly influenced by surgeon experience and case selection (19). Further work on percutaneous transforaminal endoscopic techniques has emphasized that, despite their proven efficacy, an objective learning curve exists and may affect operative duration, perioperative efficiency, and adverse event rates (19). Compared with conventional microdiscectomy, TELD may reduce hospitalization burden while preserving long-term effectiveness, but technical barriers and careful indication management continue to limit its widespread reproducibility (20). Even ambulatory transforaminal endoscopic surgery performed under awake conditions, although feasible, still requires a mature multidisciplinary team and a highly standardized workflow (21). Fleiderman et al. (22) further suggested that approximately 20 cases may be needed before the TELD learning curve is meaningfully overcome, reinforcing the notion that procedural complexity itself is a major determinant of dissemination and outcome stability. Against this background, the low complication burden and favorable long-term outcomes observed with DPAE when performed by a single team may indicate that, while preserving the minimally invasive nature of treatment, this technique may offer greater procedural accessibility and workflow reproducibility than more technically demanding full-endoscopic strategies. More importantly, the preserved safety balance suggests that the clinical advantage of DPAE is not derived from more extensive tissue manipulation, but rather from more effective interruption of key pain-generating mechanisms under conditions of limited procedural trauma. This does not imply that DPAE can replace all endoscopic or open procedures, since different techniques address different pathological spectra, decompression requirements, and structural objectives. Nevertheless, for patients whose clinical presentation is dominated by intradiscal pathology and discogenic low back pain, DPAE may represent a more balanced therapeutic option in terms of risk control, efficacy durability, and real-world implementability.

The subgroup analyses further suggested that the treatment effect of DPAE was relatively stable. Its advantage was not confined to a single clinical phenotype, but instead showed a consistent direction of benefit across strata defined by age, gender, symptom duration, degeneration grade, involved segment, and baseline pain severity, without evidence of significant interaction. This finding is noteworthy because it implies that the superiority of DPAE may not depend on selective action against one isolated pain-generating mechanism. More plausibly, it may result from simultaneous modulation of several key pathological processes involved in disk disease, thereby conferring greater robustness in heterogeneous populations such as patients with discogenic pain or contained disk herniation. Clinically, symptom generation in these patients is often shaped by multiple coexisting factors, including abnormal mechanical loading, annular fissuring, local inflammation, aberrant neural ingrowth, and endplate or adjacent structural reactions. Although patients may appear phenotypically similar, the dominant underlying pathological drivers are often not the same. When a treatment targets only a single pathway, its effectiveness is more likely to fluctuate according to baseline patient characteristics. By contrast, the consistent benefit observed with DPAE across multiple subgroups suggests that it may partially overcome the outcome variability introduced by such biological and clinical heterogeneity. Imaging follow-up studies have shown that postoperative changes in disk morphology are closely related to clinical outcome, and that the degree of intradiscal morphological restoration assessed in different reference planes can influence symptom improvement after percutaneous endoscopic procedures (23). Studies examining Modic changes and adverse outcomes after PETD have likewise indicated that the intradiscal stress environment, endplate response, and baseline imaging characteristics may continue to shape postoperative prognosis (24). Another predictive modeling study further demonstrated that BMI, calcification, symptom duration, and imaging parameters can jointly determine the risk of unfavorable outcome after PETD, highlighting that the real-world effectiveness of minimally invasive disk procedures is often the product of multiple interacting factors (25). In this setting, the persistently consistent treatment direction of DPAE across subgroups suggests that its potential benefit may not be entirely contingent upon any single baseline condition, but may instead arise from a multimodal mechanism acting on shared pain-related nodes across different pathological backgrounds, thereby buffering, at least in part, the impact of patient heterogeneity on clinical outcomes. That said, the absence of statistically significant interaction should not be interpreted as proof that true effect modification does not exist, nor does it support the conclusion that DPAE confers an identical magnitude of benefit to all patients. Given the limited sample size and the restricted statistical power of subgroup analyses, the present findings should be regarded more as a signal of robustness than as definitive evidence for precision stratification. Future studies incorporating more refined imaging markers, endplate characteristics, annular fissure morphology, and inflammatory biomarkers are needed to identify and validate the patient profiles most likely to derive benefit from DPAE.

Several limitations should also be acknowledged. First, this was a retrospective single-center cohort study. Although propensity score matching and multivariable adjustment were used to reduce confounding, residual bias cannot be completely excluded. Second, treatment allocation was not randomized, and the choice of procedure may have been influenced by both surgeon preference and patient-related factors. Third, the study lacked perioperative quantitative imaging data, inflammatory biomarker profiles, and intradiscal histopathological information; therefore, interpretation of the mechanistic advantage of DPAE remains largely inferential and clinically based rather than supported by direct biological evidence. In addition, although follow-up extended to 24 months, this duration may still be insufficient to fully characterize long-term disk degeneration trajectories and more distant reintervention risk. Future multicenter prospective randomized controlled trials incorporating quantitative MRI parameters, annular repair status, inflammatory factor profiles, and patient-reported outcomes are warranted to better define the optimal indications for DPAE, identify the patient populations most likely to benefit, and clarify its potential long-term structural protective effects.

Using a single-center real-world cohort with propensity score-matched analysis, this study systematically compared 24-month clinical outcomes between DPAE and standard Disc-FX in patients with chronic lumbar discogenic pain and contained lumbar disk herniation. The results showed that, without increasing the overall complication rate, DPAE provided superior pain relief, better functional recovery, higher rates of clinically meaningful improvement, and greater overall treatment success than Disc-FX, while also demonstrating a lower risk of reintervention. These findings suggest that, in carefully selected patients, a multimodal therapeutic strategy integrating mechanical decompression, targeted ablation, and annular modulation may control disc-related pain processes more effectively than conventional intradiscal techniques and thereby translate into more durable mid- to long-term clinical benefit.

From a clinical perspective, by simultaneously addressing abnormal intradiscal pressure, annular fissures, and the local inflammatory microenvironment through a limited-trauma access pathway, DPAE may establish a more favorable balance between structural preservation and biological modulation. As such, it may offer a minimally invasive treatment option with a more balanced trade-off between risk and efficacy for patients whose main manifestations are discogenic low back pain or contained disk herniation. Nevertheless, because the present study was retrospective and conducted at a single center, these conclusions should be interpreted cautiously and require confirmation in future multicenter prospective randomized trials. Further studies incorporating quantitative imaging markers and inflammation-related biomarkers are also needed to clarify the mechanisms of DPAE and better define the patient populations for whom it is most appropriate.

## Data Availability

The original contributions presented in the study are included in the article/Supplementary material, further inquiries can be directed to the corresponding authors.
