# Supplementary material for: Real-world 24-month pain outcomes of disk percutaneous ablation and extraction versus Disc-FX nucleoplasty for lumbar discogenic pain and contained lumbar disk herniation: a single-center retrospective cohort study
Source: Front Neurol. 2026 Jun 23;17:1839430. doi: 10.3389/fneur.2026.1839430 (PMC13337398; doi:10.3389/fneur.2026.1839430)
Supplement: Supplementary file 1 [file Table_1.DOCX]

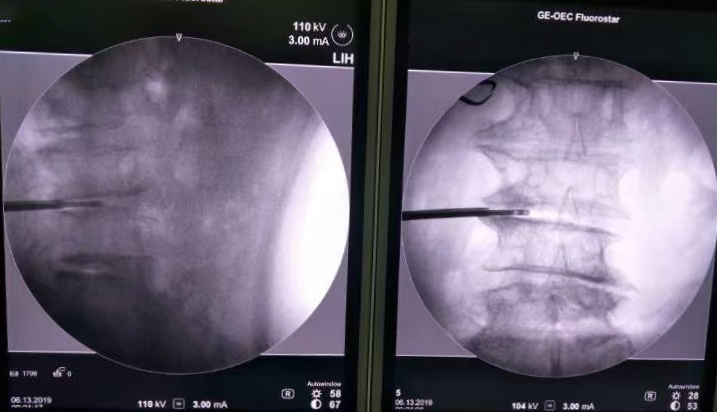


Figure S1. Representative intraoperative fluoroscopic images of DPAE.


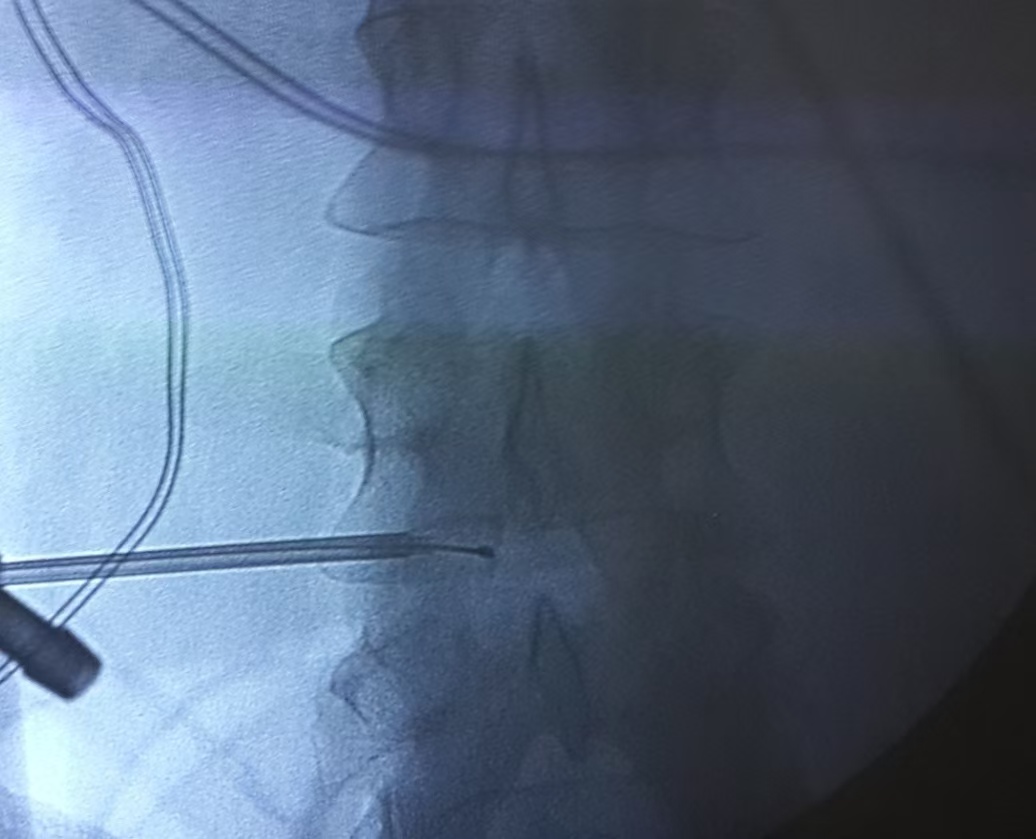


Figure S2. Fluoroscopic images confirming proper cannula and electrode placement during Disc-FX procedure.
